# Phage co-transport with hyphal-riding bacteria fuels bacterial invasion in a water-unsaturated microbial model system

**DOI:** 10.1038/s41396-021-01155-x

**Published:** 2021-12-13

**Authors:** Xin You, René Kallies, Ingolf Kühn, Matthias Schmidt, Hauke Harms, Antonis Chatzinotas, Lukas Y. Wick

**Affiliations:** 1 Helmholtz Centre for Environmental Research - UFZ, Department of Environmental Microbiology, Permoserstr. 15, 04318 Leipzig, Germany; 2 Helmholtz Centre for Environmental Research - UFZ, Department of Community Ecology, Theodor-Lieser-Str. 4, 06120 Halle, Germany; 3 Institute of Biology/Geobotany and Botanical Garden, Martin Luther University Halle‐Wittenberg, Halle, Germany; 4 German Centre for Integrative Biodiversity Research (iDiv) Halle-Jena-Leipzig, Deutscher Platz 5e, 04103 Leipzig, Germany; 5 Helmholtz Centre for Environmental Research - UFZ, Department of Isotope Biogeochemistry, Permoserstr. 15, 04318 Leipzig, Germany; 6 Institute of Biology, Leipzig University, Talstr. 33, Leipzig, 04103 Germany

**Keywords:** Microbial ecology, Ecosystem ecology, Macroecology, Population dynamics

## Abstract

Nonmotile microorganisms often enter new habitats by co-transport with motile microorganisms. Here, we report that also lytic phages can co-transport with hyphal-riding bacteria and facilitate bacterial colonization of a new habitat. This is comparable to the concept of biological invasions in macroecology. In analogy to invasion frameworks in plant and animal ecology, we tailored spatially organized, water-unsaturated model microcosms using hyphae of *Pythium ultimum* as invasion paths and flagellated soil-bacterium *Pseudomonas putida* KT2440 as carrier for co-transport of *Escherichia* virus T4. *P. putida* KT2440 efficiently dispersed along *P. ultimum* to new habitats and dispatched T4 phages across air gaps transporting ≈0.6 phages bacteria^−1^. No T4 displacement along hyphae was observed in the absence of carrier bacteria. If *E. coli* occupied the new habitat, T4 co-transport fueled the fitness of invading *P. putida* KT2440, while the absence of phage co-transport led to poor colonization followed by extinction. Our data emphasize the importance of hyphal transport of bacteria and associated phages in regulating fitness and composition of microbial populations in water-unsaturated systems. As such co-transport seems analogous to macroecological invasion processes, hyphosphere systems with motile bacteria and co-transported phages could be useful models for testing hypotheses in invasion ecology.

## Introduction

To cope with the heterogeneous and highly changeable soil environment, microorganisms have evolved inter-microbial co-transport strategies to gain motility and colonize new habitats (reviewed by [1]). For instance, bacteria have been found to efficiently disperse along hyphae in soil [2–4]. Fungi embody up to 75% of the subsurface microbial biomass [5]. Their hyphae create fractal-like mycelial networks of 10^2^–10^4^ m g^−1^ of topsoil and efficiently spread in heterogeneous habitats, penetrate air-water interfaces and cross over air-filled pores [5–7]. They thereby serve as pathways (also called “fungal highways” [2]) for bacteria to efficiently disperse, forage [8] and colonize new habitats [4, 9]. However, hyphae may reduce dispersal of intrinsically nonmotile yet abundant (>10^8^ g^−1^ soil [10]) soil virus-like particles as they have been shown to retain waterborne phages [11, 12]. Considering the slow diffusion (~0.034 mm/d [13]) and enhanced inactivation at dry conditions [14, 15], transport of phages seems particularly restricted in water-unsaturated habitats. Recent studies have revealed that phages in aquatic environments can adsorb to surfaces [16, 17], mucus [18], flagella [19] of non-host bacteria or even sheath surrounding them [20]. As hyphae are adapted to water-unsaturated habitats we here hypothesize that hyphae allow for phage co-transport with hyphal-riding bacteria and thereby fuel the fitness of invading bacteria in new regions (alien ranges) of water-unsaturated habitats. Co-transported phages, which are not detrimental to the carrier bacteria but specific to resident bacteria in alien ranges thereby may serve as “biological weapons” [21, 22] and increase the competitive ability and fitness of the invading carrier bacteria. Temperate phages integrated in bacterial genome (i.e. as prophages) have been suggested to serve as agents of “bacterial warfare” [22–24]. In aquatic environments, lytic phages adsorbed to bacteria have been shown to facilitate phage infection of biofilm bacteria and to promote biofilm colonization by carrier bacteria [19]. In unsaturated environments like soil, however, little is known about co-transport of phage with motile bacteria and the associated effect on bacterial population dynamics.

In analogy to the previously published MAcroecological Framework of Invasive Aliens (MAFIA [25]) we here explored the possibility to use spatially organized microcosm systems to mimic the stages of the invasion process (i.e. transport, introduction, establishment, and spread) [25–28] of co-transported phages and bacteria in the hyphosphere. Unlike biological invasions in macroecological systems, our model system bears the advantage that all interacting species, their locations and location characteristics as well as invasion events are known and can be manipulated. In our model the “native range” and the “invaded” or “alien range” are two agar patches that are separated by an air gap. The air gap serves as a barrier that only can be overcome by bacterial movement along hyphae as invasion pathways crossing the native and alien ranges. Such water-unsaturated systems allow (i) to evaluate the transport efficiency of flagellated and non-flagellated bacteria as vectors to transport phages into alien ranges and (ii) to quantify possible population effects of co-transported phages in the alien range. Our approach revealed that motile bacteria can help phages to migrate into water-unsaturated habitats and that phage co-transport can fuel the settlement and fitness of hyphal-riding bacteria in host pre-colonized alien ranges.

## Materials and methods

### Strains, growth conditions, and enumeration methods

**Fig. 1:**
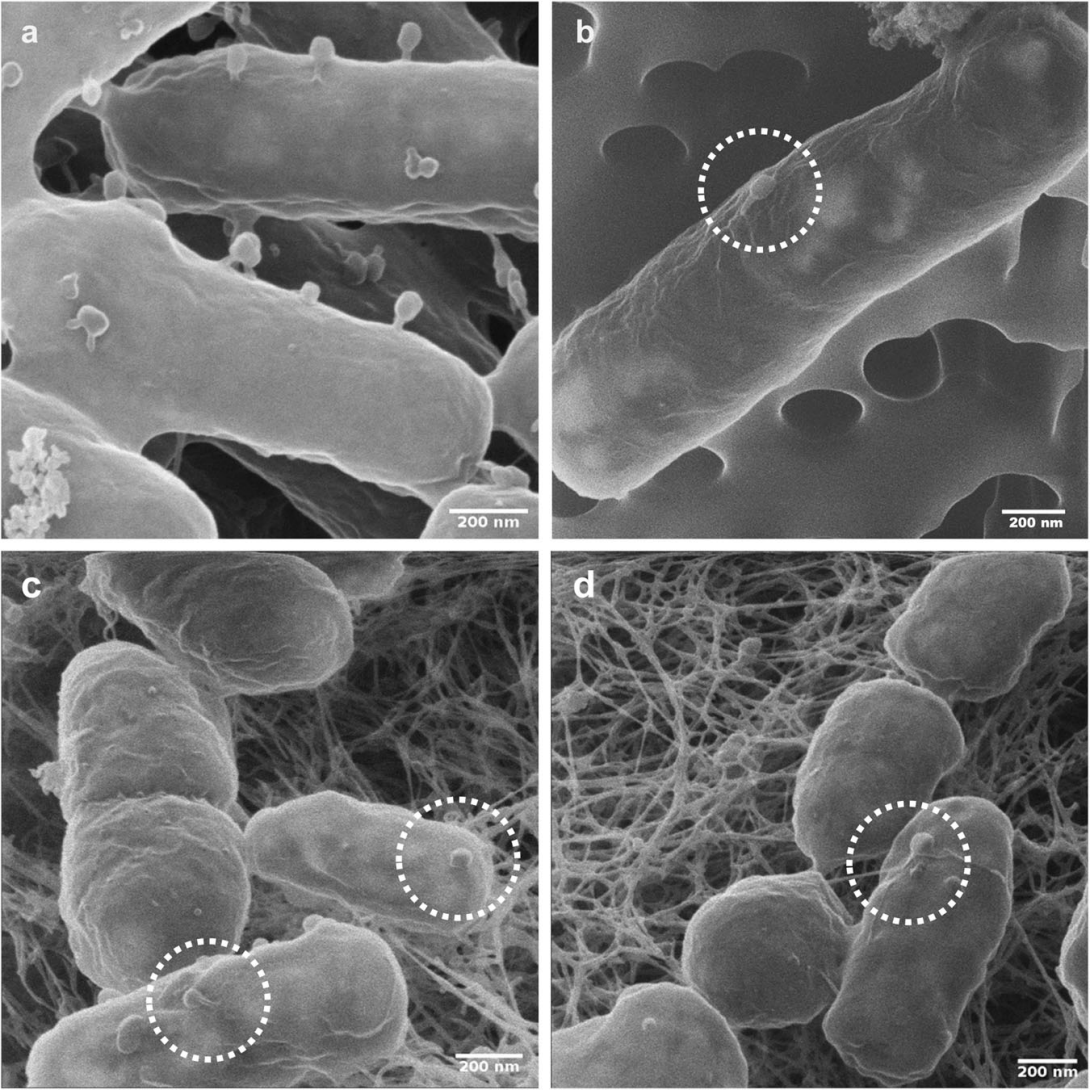
Helium ion microscopy (HIM) visualization of T4 phage adsorption to *E. coli* host and non-host *P. putida* KT2440 cells. **a**, **b** visualize cells from phage adsorption experiments (cf. materials and methods) reflecting tail-mediated adsorption to *E. coli* host cells (**a**) and capsid-driven adsorption to non-host *P. putida* KT2440 (**b**). **c**, **d** visualize tail- (**c**) and capsid-driven (**d**) phage adsorption to biofilm cells growing on agar patch B on day 2 in experiments evaluating population effect of T4 co-transport with *P. putida* KT2440 (cf. Fig. 3).

GFP-labeled wild type [29] soil-bacterium *Pseudomonas putida* KT2440 (termed hereafter as WT) and its non-flagellated mutant Δ*filM* were used as carriers for phage co-transport. Δ*filM* was obtained by allelic exchange with a truncated version of *filM* [30] and used to test the role of flagella for phage adsorption and phage co-transport. Both strains were kindly provided by Arnaud Dechesne (DTU, Denmark). They were cultivated in LB medium on a gyratory shaker at 30 °C and 150 rpm. For microcosm experiments, an overnight culture (OD_600_ ≈ 2) was washed once with PBS buffer (100 mM) and adjusted to reach an OD_600_ ≈ 4 (≈8 × 10^6^ cell µL^−1^). Hyphae of the oomycete *Pythium ultimum* [31] were used as model dispersal networks, as *P. ultimum* is fast growing and provides hydrophilic hyphal surfaces allowing for efficient bacterial dispersal [2]. It was pre-grown on potato dextrose agar (PDA) at room temperature (RT) [32]. *Escherichia* virus T4 (T4) was selected in co-transport experiments, because it is a commonly used model phage and is known to adsorb to cell surfaces [33]. T4 was propagated on its host *E.* *coli* (Migula 1895) using the liquid broth method in DSM544 medium [35]. T4 and *E. coli* (Fig. 1a) were purchased from Deutsche Sammlung von Mikroorganismen und Zellkulturen GmbH (DSMZ, Braunschweig, Germany). *E. coli* was cultivated in DSM544 medium at RT on gyratory shaker at 150 rpm (generation time = 41 ± 0.1 (SD) min in the exponential phase). For microcosm experiments, 10 µL of an overnight *E. coli* culture were transferred into 20 mL fresh DSM544 medium and cultivated at 30 °C until early exponential growth (OD_600_ ≈ 0.4).

Enumeration of *E. coli* and *P. putida* KT2440 were carried out by counting colony forming units (CFU) on LB agar incubated at 30 °C overnight. When both strains were present in a sample, CFU of GFP-tagged *P. putida* KT2440 were counted with an epifluorescence microscope equipped with a black-and-white camera (AZ 100 Multizoom; Nikon, Amsterdam, Netherlands) under the GFP channel using NIS Elements software. Plaque forming unit (PFU) enumeration was done using a modified small-drop plaque assay technique as detailed earlier [36] allowing the double-layer counting plates to be incubated overnight at 30 °C. The whole-plate plaque assay (cf. [37]) was also performed to crosscheck PFU counts for samples with zero PFU count by small-drop plaque assay.

### Determination of phage adsorption to bacteria

Adsorption efficiencies of T4 to WT, Δ*filM* and *E. coli* were quantified at phage-to-bacteria ratios of 1, 0.1, and 0.01 in 6–8 replicates as described earlier [38]. In brief, suspensions of bacteria (≈10^8^ CFU mL^−1^) and T4 were incubated in PBS at RT for 1 h (15 min for T4 and *E. coli*) and centrifuged at 8000 × *g* at 4 °C to pellet bacteria and adsorbed phages. Amounts of adsorbed phages were estimated by the loss of free phages after centrifugation; i.e. phage adsorption (%) calculated by the ratio of adsorbed phages to total phages prior to centrifugation. A phage-only control was also included to determine the stability and change of infectivity of T4 in the medium and during centrifugation.

### Quantification of phage co-transport by hyphal-riding bacterial carriers

**Fig. 2:**
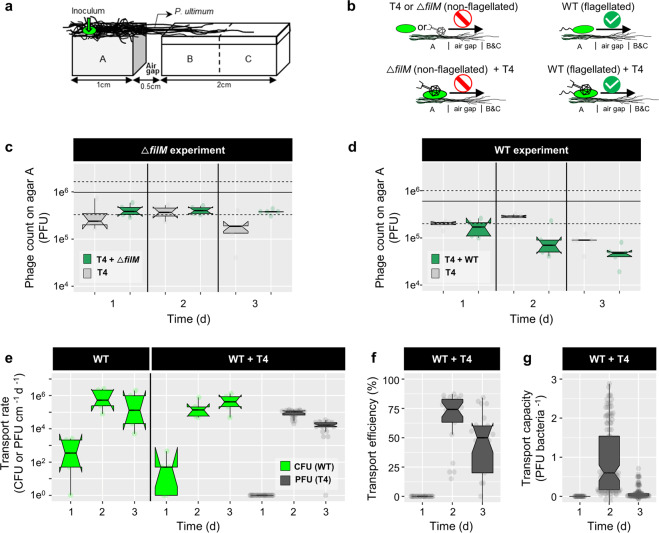
Co-transport of T4 with *P. putida* KT2440 (WT or non-flagellated Δ*filM*) along hyphae of *P. ultimum*. **a** Scheme of the microcosm setup specifying microbial inoculation points and the spatial arrangement and dimensions of agar patches A, B, and C. **b** Experimental scenarios and observed results. Upper left panel: T4 or Δ*filM* do not disperse along hyphae over the air-gap. Lower left panel: Δ*filM* does not transport T4 along hyphae. Upper right panel: WT disperse along hyphae. Lower right panel: WT disperses along hyphae and transports T4 along hyphae over the air-gap. **c**, **d** T4 counts on agar patch A after 1 day in the absence and presence of Δ*filM* (**c**) and WT (**d**). The solid and dashed lines indicate the median of 5 replicates and its 95CI of the initial inocula. **e** Time-dependent cumulative transport rates of WT (R_WT_, in green) and phages (*R*_T4_, in gray) to agar patches B and C (cf. Eq. 1). **f** Time-dependent phage transport efficiency in presence of WT (*E*_T4_). **g** Time-dependent phage transport capacity of WT (*C*_T4_). Data on T4 transport by Δ*filM* are not shown as no transport was observed. Notches of the boxes represent 95CI of 5 replicates. If notches of two conditions do not overlap, it indicates a statistical difference between the two conditions.

**Fig. 3:**
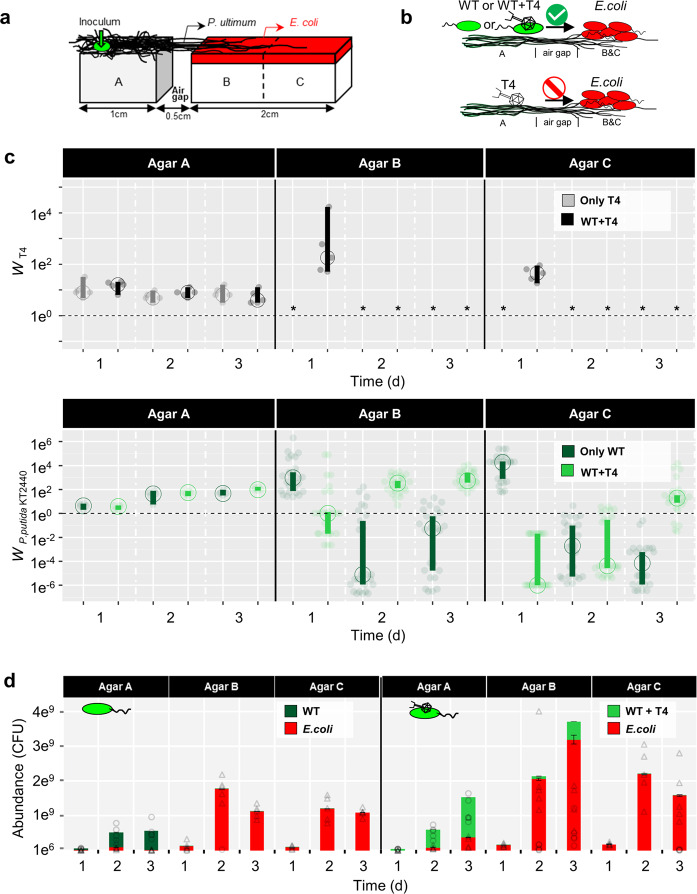
Effects of T4 co-transport with flagellated *P. putida* KT2440 (WT) along hyphae of *P. ultimum* phage, *E. coli* and WT counts on agar patches A–C. **a** Scheme of the microcosm setup specifying microbial inoculation points and pre-colonized *E. coli* on agar patches A, B, and C. **b** Overview of the scenarios for evaluating invasion. Upper panel: T4 co-transports with WT along hyphae to an *E. coli* covered alien range (i.e. agar patches B and C). WT disperses along hyphae to the *E. coli* pre-colonized alien range. Lower panel: T4 is not able to disperse along hyphae to the alien range. **c** Time-dependent absolute fitness of T4 (*W*_T4_) and of *P. putida* KT2440 (*W*_WT_) on agar patches A, B, and C in presence and absence of phage-bacterial co-transport. Dashed line represents no change of the population size (*W*_i_ = 1) and asterisk indicates that no PFU were detected. The vertical bars represent 95CI calculated from 5 biological replicates. If vertical bars of two conditions do not overlap, it indicates a statistical difference between the two conditions. **d** Abundance of *E. coli* and WT populations on agar patches A, B, and C with and without T4 co-transport (triangles and circles represented individual data points of *E. coli* and *P. putida* KT2440 in 5 replicates). Please note that abundances of WT < 10^8^ CFU (e.g. observed in the case of WT + T4” on 3d) are not visible due to the plot scale.

T4 co-transport with carrier bacteria was quantified in quintuplicate laboratory microcosms mimicking unsaturated (i.e. vadose) soil zones in the presence and absence of hyphal networks (Fig. 2a). The microcosms consisted of an agar patch A (PDA, 2% agar (w/v), l × w × h = 1 × 1 × 0.6 cm) that was separated from an agar strip (l × w × h = 2 × 1 × 0.6 cm) by a 0.5 cm air gap. *P. ultimum* was pre-inoculated on agar patch A for 3–4 days to reach >0.5 cm hyphal length prior to finally assembling the microcosms. The agar strip (that was split into equally sized patches B and C before harvesting, Figs. 2a and 3a) however was freshly prepared upon setting up the microcosm. It was made from minimal medium agar (MMA) to avoid bacterial growth and consisted of a top layer (MMA, 0.6% agar (w/v), h = 0.1 cm) and a bottom layer (MMA, 2% agar (w/v), h = 0.5 cm). All agar patches were placed in sterile Petri dishes. To analyze the transport of T4 along hyphae of *P. ultimum* in presence and absence of carrier bacteria, five different scenarios were used (Fig. 2b): (i) WT, (ii) WT + T4, (iii) T4, (iv) Δ*filM*, and (v) Δ*filM* + T4. *P. ultimum* pre-grown agar patches (equal size as agar patch A) with T4 or WT + T4 were used to quantify hyphal effects on T4 infectivity. Inactivation of T4 on agar surfaces was studied on agar patches in the absence of *P. ultimum*. In scenarios (ii) and (v) bacteria with previously adsorbed phages were added. To do so, T4 (6 × 10^9^ PFU mL^−1^) was co-incubated in PBS with WT or Δ*filM* (≈8 × 10^9^ cells mL^−1^) at RT for 1 h at 125 rpm and then centrifuged (8000 × *g* for 10 min at 4 °C) to discard free phages in the supernatant. We used a phage-to-bacteria ratio of ≈1, because phage adsorption to bacteria was observed highest at this ratio (Fig. S1a). The remaining pellet containing bacteria and adsorbed phages was washed once and concentrated by re-suspension in PBS to reach an inoculum density of OD_600_ (estimation) ≈20. Inocula with either bacterial cells (≈4 × 10^10^ cells mL^−1^) or phages (≈6 × 10^8^ PFU mL^−1^) in PBS served as controls. 1 µL of the respective inoculum (i.e. ≈4 × 10^7^ bacteria or 6 × 10^5^ phages) was placed at 0.25 cm from the left edge of agar patch A. After inoculation the Petri dishes were sealed with Parafilm, placed in a plastic container and incubated at 20 °C in the dark. After 24, 48, and 72 h the microcosms were sacrificed and phage and bacteria numbers were quantified on agar patches A, B, and C by PFU and CFU. Isolation of phage and bacteria from agar was done as described previously [8]; i.e. cut agar pieces were suspended in 3 mL PBS in glass tubes, vortexed at maximal speed for 1 min and then sonicated (2 × 30 s with a break of 1 min). Phage-bacteria suspensions were 1:1 extracted with chloroform in order to inactivate and remove bacterial biomass prior to PFU quantification.

### Evaluation of population effects due to phage co-transport with hyphal-riding bacteria

Effects of phage co-transport on the populations of carrier bacteria invading an alien range occupied by competing bacteria were evaluated in five replicates in similar microcosms as described above. The double-layer of agar patches B and C, however, contained nutrient agar (DSM544) and the upper agar layer was densely populated by *E. coli* (Fig. 3a). Prior to the experiment, the thin upper agar layer of agar patches B and C was inoculated with *E. coli* (5 ± 0.5 (SD) × 10^4^ CFU cm^−2^) and allowed to grow at RT for 3 h (≈4 generations, 2.3 ± 0.1 (SD) × 10^5^ CFU cm^−2^). Three different scenarios were studied using either (i) T4, (ii) WT, or (iii) WT + T4 as inocula. After 24, 48, and 72 h, all agar patches A, B, and C were harvested and phages and bacteria quantified as described above.

### Helium ion microscope imaging

Helium ion microscope (HIM) imaging was performed with suspensions from phage-bacteria adsorption assays and surfaces of agar patches B after invasion of phage-carrying WT *P. putida* KT2440 into *E. coli* populations. HIM imaging of surfaces of T4 plaques on a lawn of *E. coli* served as control. Suspension containing phage-bacteria associations from the adsorption assays were mixed at a ratio of 1:1 with 2% (v/v) paraformaldehyde in 0.2 M sodium‐cacodylate buffer (pH = 7.4) and allowed to stand for 2 h for chemical fixation. The suspension was then transferred onto 0.22-µm pore‐size polycarbonate filter papers (Merck‐Millipore) using a Sartorius hand‐filtration device. The filter papers were rinsed twice for 5 min with Na‐cacodylate buffer to remove salts and debris. The samples were dehydrated in a graded aqueous ethanol series (30, 50, 70, 80, 90, and 100% EtOH) and critical point dried. To observe phage-bacteria associations on agar surfaces, the agar patches were submersed in Petri-dishes in the fixative for 2 h (cf. above). The fixative was then gradually exchanged using a graded aqueous ethanol series (>50% fixative replacement each time to avoid material loss) and the sample finally critical point dried. The dried samples were mounted onto standard stubs for electron microscopy using a conductive silver epoxy glue and imaged by a Zeiss Orion NanoFab (Zeiss, Peabody, MA, USA) scanning HIM using an ion-landing-energy of 25 keV, a 10-µm aperture and an Everhard–Thornley-type secondary electron detector. To achieve both high lateral resolution (≤2 nm) and contrast, the beam current was set between 0.08 pA (high magnification) and 0.25 pA. Charge compensation during imaging was achieved with an electron flood-gun operated in line-flooding mode. In order to avoid beam damage and to allow for efficient charge compensation the dwell time of the beam on a pixel was kept between 0.5 and 1.0 µs.

### Data analysis and statistics

The time-dependent transport rate (*R*_i_; shown in Eq.1) of phages (*R*_p_, PFU cm^−1^ d^−1^) or bacteria (*R*_b_, CFU cm^−1^ d^−1^) was obtained by normalizing the number of phages transported (*N*_p_, PFU) or bacteria transported (*N*_b_, CFU) to the dispersal distance (*d*, cm) and the time (*t*, d) until harvesting. Because T4 got rapidly inactivated on agar surfaces (99% loss of PFU in <24 h, Fig. S2a) and subsequent low phage numbers were elusive to direct quantification, *N*_p_ was approximated by the difference of phages counted at the point of inoculation (i.e. agar patch A) in the absence and presence of carrier bacteria. This approach was possible as the presence of either *P. ultimum* or *P. ultimum* and *P. putida* KT2440 co-cultures did not influence T4 infectivity and enumeration (Fig. S2c).

1 $$R_i = \frac{{N_{{{{{{{\mathrm{i}}}}}}}}}}{{{{{{{{{\mathrm{d}}}}}}}} \times {{{{{{{\mathrm{t}}}}}}}}}}$$

For co-transported phages, time-dependent transport capacity (*C*_p_, PFU bacteria^−1^) reflect the average number of phages transported by a single bacterium and transport efficiency (*E*_p_, %) the fraction of phages dispatched by carrier bacteria. For calculation details, please refer to extended materials and methods in SI.

To evaluate the effect of phage co-transport on bacterial or phage populations at time *t*, the absolute fitness (*W*_i_) [39] of a given population i was calculated using Eq. 2. *W*_i_ is the time-dependent ratio of the population size (bacteria or phages) on given agar patches in the presence of *E. coli* (N^*^_i_, CFU or PFU) and absence of *E. coli* (*N*_i_, CFU or PFU). *W* > 1 and *W* < 1 indicate an increase and a decrease of the population size, while *W* = 0 refers to population extinction (c.f. extended materials and methods in SI for calculation details).

2 $$W_{{{{{{{\mathrm{i}}}}}}}} = \frac{{N_{{{{{{{\mathrm{i}}}}}}}}^ \ast }}{{N_{{{{{{{\mathrm{i}}}}}}}}}}$$

Data were plotted as transparent dots and statistics were displayed as median (circle) with 95% confidential interval (95CI, vertical bar) or boxplot notches as modified from [40]. The 95CI was determined by bootstrapping (1000 samples) and derived from the 2.5th and 97.5th percentile [41]. When the 95CIs of two conditions (*n* ≥ 5) do not overlap, it indicates a statistical difference between these two conditions [42].

## Results

### Adsorption of T4 phage to WT and ΔfilM

Being a prerequisite for phage co-transport with carrier bacteria we tested the adsorption of T4 phages to the flagellated WT of *P. putida* KT2440 and its non-flagellated Δ*filM* mutant. We found that 13–62% of T4 particles adsorbed to the non-host WT with the highest adsorption (62%) observed at a phage to bacteria ratio of 1 (Fig. S1a). No statistically significant differences between T4 adsorption to flagellated WT and Δ*filM* were observed at this phage to bacteria ratio (Fig. S1b). Adsorption to WT was thus consistently lower and more variable than to the host strain *E. coli* (68–84%; Fig. S1a). T4 adsorption was further evidenced by HIM visualization. It revealed capsid-driven adsorption of T4 (Fig. S1d, e) to the surface of *P. putida* KT2440 leaving the phages’ tails unattached (Fig. 1b). This is in contrast to T4 adsorption to host *E. coli*, where perpendicular adsorption with phage tails bound to bacterial surfaces was found (Figs. 1a and S1c).

### Effect of hyphal-riding bacteria on phage co-transport

In analogy to the different stages of the plant or animal invasion processes [25, 26] we developed a spatially organized microcosm system to evaluate phage co-transport with hyphal-riding bacteria invading new habitats (i.e. agar patches B and C; Fig. 2a). To challenge the role of bacterial motility for phage co-transport, we used flagellated WT and the non-flagellated Δ*filM* mutant in five different scenarios (Fig. 2b): (i) T4, (ii) Δ*filM*, (iii) WT, (iv) Δ*filM* + T4 and (v) WT + T4. No airborne transport of phages was observed in the microcosms (Fig. S2b). As T4 infectivity got rapidly lost on agar surfaces (>99% loss within 24 h, Fig. S2a) preventing reliable enumeration on agar patches B and C, phage transport rates (*R*_p_; Eq. 1) were calculated by differences of phage counts on agar patch A in all scenarios. Contrary to our observation on sterile agar surfaces (Fig. S2a) T4 maintained its infectivity up to 2 days when placed on agar patches covered by *P. ultimum* (Fig. 1c). No reduction in T4 counts in the absence of bacteria or in presence of Δ*filM* was observed over 2 days pointing at negligible diffusion of viable phages or phage co-transport by Δ*filM*. Similarly, inoculation of WT + T4 on isolated *P. ultimun* agar (i.e. no WT exportation; Fig. S2c) showed no reduction in T4 counts (Fig. S2c). By contrast, inoculation of WT + T4 on interlinked *P. ultimum* agar (i.e. WT exportation allowed; Fig. 2a) resulted in a significant (>65%) reduction of T4 counts after 2 days and a transport efficiency of *E*_p_ ≈ 60% (Fig. 2e). The phage transport rates (*R*_p_ ≈ 10^5^ PFU cm^−1^ d^−1^, Fig. 1d) thereby coincided with hyphal dispersal rates of the WT (*R*_b_ ≈ 1.4 × 10^5^ CFU cm^−1^ d^−1^; Fig. 1d) suggesting an apparent transport capacity of *C*_p_ ≈ 0.6 PFU bacteria ^−1^ after 2 days (Fig. 1g). After 3 days a 10-fold decreased T4 transport rate (*R*_p_ ≈ 1.4 × 10^4^ PFU cm^−1^ d^−1^, Fig. 1d) and a 20-fold reduced apparent T4 transport capacity (*C*_p_ ≈ 0.03 PFU bacteria^−1^) were observed. This is likely due to growth of WT bacteria and/or the inability of the WT progeny on agar patch A to get into contact with T4 phages. Transport rates of WT bacteria were similar regardless of the presence of T4 (Fig. 2e).

### Effects of phage co-transport on bacterial invasion and invader fitness

To evaluate the community effect of T4 co-transport with hyphal-riding WT, agar patches B and C were covered with 2.3 ± 0.1 × 10^5^ CFU cm^−2^ of *E. coli* as local host bacteria of T4 (Fig. 3a). Development of T4, WT, and *E. coli* counts was quantified over time in four different scenarios (cf. Fig. 3b): (i) PBS only, (ii) WT, (iii) T4 and (iv) T4 + WT. In the absence of WT, no diffusion of infectious T4 along hyphae to agar patches B and C was observed at any time (Fig. 3c). In presence of WT, however, ∼10^5^ and ~10^4^ PFU were recovered from agar patches B and C after 1 day leading to a 45–180-fold increased phage abundance (Fig. 3c; 45 < *W*_T4_ < 180 as defined by Eq. 2). The presence of T4 went along with HIM-detectable bacterial lysis of *E. coli* (Fig. S3e) indicating previous phage lysis on day 1. No T4, however, were detected on day 2 (Fig. 3c) despite HIM-detectable phages adsorbing to bacterial surfaces either in tail-mediated perpendicular (Fig. 1c) or capsid-mediated coaxial positions (Fig. 1d). Tail-mediated adsorption thereby points at an infection of host cells (Fig. 1a) while capsid-mediated adsorption may refer to unspecific interaction with non-host cells (Fig. 1b). In the absence of T4 the carrier WT cells invaded and established on agar patches B and C in the first 24 h (*W*_WT_ > 930) accounting for 0.08–0.39% of all bacteria. Thereafter WT got strongly inhibited (*W*_WT_ < 0.06) and comprised less than 0.01% of the population; this, despite a constantly growing WT population on agar patch A (*W*_WT_ > 1) and a likely on-going WT invasion from agar patch A (Fig. 3c).

By contrast, T4 co-transport with WT promoted invasion and fitness of the carrier bacteria after a delay of 1 day: on agar patch B the absolute fitness of WT increased from *W*_WT_ ≤ 1 (t = 1 d) to *W*_WT_ = 556 (≈5 × 10^8^ CFU) at t = 3 d (Fig. 3c). On agar patch C the WT fitness changed from *W*_WT_ < 1 (t = 1–2 d) to *W*_WT_ > 1 after 3 days. After 3 days WT accounted for ≈14% and 1% of the total bacterial population on agar patches B and C, resp. (Fig. 3d). Epifluorescence microscopy analysis thereby revealed that gfp-labeled WT established best in the vicinity of the hyphae (Fig. S3b, c), suggesting highest phage-clearance effects on resident *E. coli* in the hyphosphere. Invasion of WT, however, had no negative impact on the abundances of *E. coli* cells in agar patches B and C (Fig. 3d). We further checked the effect of phage co-transport on overall phage counts and abundances of host and carrier bacteria in the microcosms (i.e. agar patches A, B, and C) (Fig. S4). While co-transport did not influence T4 abundances (Fig. S4a), it clearly enhanced the abundance of WT (Fig. S4b), yet not influenced the *E. coli* abundances (Fig. S4c).

## Discussion

### Phage co-transport with hyphal-riding bacteria

Although described for aquatic environments [19, 43, 44], little is known on viral co-transport with non-host microorganisms in vadose habitats; even though adsorption and subsurface co-transport of nano-colloids with organic or biological materials has been described nearly two decades ago [45]. Viral deposition fluxes though the atmospheric boundary layer of up to 7 × 10^9^ m^−2^ d^−1^ [46] or event-driven transport of nano-colloids and viruses of up to 1 × 10^10^ particles mL^−1^ seepage water [47] suggest frequent collision of viruses with subsurface biological materials in vadose habitats. Here, we show that *Escherichia* virus T4 can adsorb to the hyphal-riding soil-bacterium *P. putida* KT2440 (Fig. S1). It thereby gets efficiently dispersed in water-unsaturated environments (Fig. 2) and invades new habitats (Figs. 2a and 3) even if this involves crossing air-filled spaces (Figs. 2b and 3d). No diffusion of T4 along hyphae of *P. ultimum* and no T4 co-transport with the non-flagellated Δ*filM* was detected. Flagellar mobility hence seems to be a driver for efficient phage transport by bacteria. Although a few studies have reported on preferential adsorption of phages to bacterial flagella [19], we found similar efficiencies of T4 adsorption to flagellated WT and non-flagellated Δ*filM* (Fig. S1b). T4 adsorption to the bacterial cell surface rather than to flagella was also confirmed by HIM imaging. Microscopy suggested that capsid-mediated sorption to cells left the phage tails unattached. This allows adsorbed phages to remain infectious, i.e. transferable to thermodynamically favored adsorption to host cells [48]. We chose T4 as model phage as it is known to adsorb to cell surfaces and to be less adsorptive than smaller phages [33, 34]. T4 adsorption to and co-transport by carrier bacteria hence may lead to conservative estimates for phage adsorption and co-transport. For instance, a recent study using *Bacillus cereus* as a model bacterium for waste water has shown its ability to adsorb phages of different morphologies from 10 viral families [19]. T4, furthermore, was also found to adsorb to the soil-bacterium *P. fluorescens* LP6a despite of its highly distinct physico-chemical surface properties [49] from *P. putida* KT2440 (Fig. S1a). As hyphal-riding bacteria are widespread in many unsaturated environments (e.g. bulk soil [3, 50, 51], the vicinity of roots [52, 53] and cheese rinds [54]), we hence speculate that other combinations of phages with hyphal-riding carrier bacteria may also lead to phage co-transport. As phage co-transport with non-host bacteria can take place over days (Fig. 2) one may speculate that such co-transport may be more efficient than recently reported co-transport of phages with their host cells (“virocells”) during the (typically small, e.g. ≥20 min for T4) latent period [55].

### Benefits of phage co-transport for bacterial carriers

**Fig. 4:**
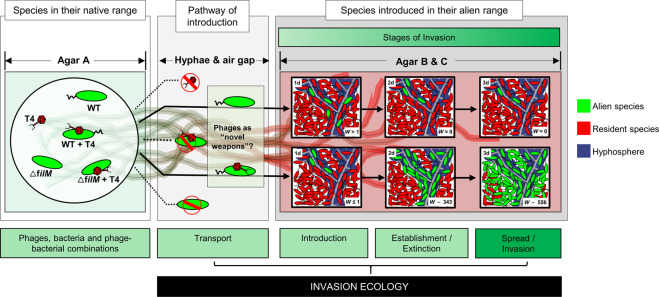
Equivalence between the MAcroecological Framework of Invasive Aliens (MAFIA) and the microsphere model system used in this study (cf. Fig. 3a). Based on the background sketch of the microsphere model system, the main findings of the study are summarized and illustrated in the flow chart. Boxes and contents are directly mirrored from the recently published MAcroecological Framework of Invasive Aliens [25] built upon [26–28].

Our data show that T4 co-transport fosters fitness of hyphal-riding WT cells if invading alien ranges occupied by resident T4 host cells (Figs. 3 and 4). Over 48 h the invasion and colonization of WT occurred in close vicinity (i.e. in the hyphosphere) of colonizing hyphae acting as preferential transport pathways for bacteria carrying adsorbed lytic phages (Fig. 4). Co-transported phages provided significant fitness gain for carrier bacteria in the alien range after 1 day (Fig. 3c). Yet, no phages could be detected at later stages although HIM analyses clearly revealed both phage particles adsorbing to bacterial surfaces (Fig. 1c, d) and lysed *E. coli* cells (Fig. S3e) in the hyphosphere (Fig. S3b). Such increase of the T4 abundance during initial WT carrier invasion may have been promoted by high accessibility of *E. coli* cells to T4 introduced by hyphal-riding WT. Lysis of host cells coupled with exponential growth of carrier WT cells in the hyphosphere however may have reduced *E. coli* cell density, and, hence, T4 accessibility and subsequent host infection. Similar phenomenon has been reported in spatially organized biofilms with two *Pseudomonas* strains, where the growth of the phage insensitive strain largely reduced the phage abundance by blocking their access to their host [56]. Likewise, on-going T4 transport may have triggered antiphage mechanisms in the *E. coli* biofilms [57, 58]. Stationary *E. coli* (i.e. after 1d, Fig. 3d) are well-known to inhibit T4 infection by adsorbing phage particles without producing viral progenies for weeks [59–63]. Such inhibitions has been described for T4, yet not for other phages (e.g. T7 can indefinitely infect stationary *E. coli* [64]). We thus believe the undetectable T4 (after 1d) is a special case in this study and cannot be generalized for other phages. Our data indicate that initial WT invasion in the absence of T4 was more efficient (*W*_WT_ > 930) than in its presence (*W*_WT_ ≈ 1). As transport rates of WT and WT + T4 did not differ (Fig. 2e, *P* > 0.05), we speculate that phage infection may lead to growth inhibition of WT carrier cells as demonstrated in a mix-culture of *Enterococcus faecalis* [65]. Hyphal-riding invader WT cells however could not establish in the absence of T4 and—likely due to higher fitness of resident *E. coli*—got eliminated after initial colonization (Figs. 3c, d and 4). Hyphae thereby served as dispersal pathways allowing for access to and competition of T4-carrying WT with *E. coli* in the alien range. While hyphae had been described as scaffolds for microbial transport [4] and evolution [66], less is known on their role as drivers for phage transport and bacterial invasion and competition. Diffusion of viral particles is extremely slow (~0.03 mm d^−1^) [13, 67] and mostly hampered when facing air gaps (e.g. macropores). In such unsaturated environment, hyphae is the indispensable dispersal network, which bridges zones separated by air gaps and enables efficient co-transport of phages and bacteria (e.g. >2.5 mmd^−1^ in this study). As demonstrated in our model system that fungi can act as corridor for phage-bacterial co-transport, future work needs to challenge the ability of hyphae to transport unknown phage-bacterial pairs in a soil realm or to use hyphae as selective pathway to isolate co-transporting phage-bacterial pairs.

### Microbial co-transport as a model for studying biological invasion

Biological invasion experiments are frequently impossible [68] or even unethical, due to the harm they can produce [69]. Hence, conceptual models (e.g. frameworks) exist to provide a better overview to explain and predict biological invasions and to provide a testing bed for hypotheses. Here we tailored a well-controllable model ecosystem in analogy to the MAFIA [25, 26] to study transport, introduction, establishment and spread of hyphal-riding bacteria in presence and absence of co-transported phages (Fig. 4). We see the potential of our model system as an in-vitro testing bed for specific mechanisms proposed in the biological invasion frameworks (Fig. 4). Due to its high degree of controllability and based on extended knowledge on bacterial-fungal interactions [2, 3, 8, 54], phage sorption to bacteria [18, 19, 44], or the role of phages promoters of bacterial colonization [19, 22, 70] it may be manipulated in order to simulate real-world situations at micro-scale. For example, our findings resemble the concept of “novel weapon” in plant ecology (e.g. biochemical possessed by invading species that is fatal to resident species) [21] and are analogous to the spread of infectious disease in animal ecology (e.g. grey squirrel being vectors for squirrel pox infecting European red squirrels [71]). Many elements in our model simulate real-world situations at micro-scale. For example, the biogeographic barriers (e.g. oceans or mountain ranges) [72] can be represented by the air gap (in our model) or other material (e.g. heavy metals) that prevents the bacterial dispersal to agar B (or the alien range, in biological invasions terminology, Fig. 4) from agar A (the native range). To test for the efficiency of different pathways, such as shipping routes [73, 74] in the real world, we can manipulate the volume of the hyphae or their physical properties (e.g. hydrophilic hyphae are more efficient in transporting bacteria than hydrophobic ones) [2]. Similarly, many other parameters can be manipulated in our system (see an overview and references for further examples in Fig. S5). Please note, however, we do not expect that our model system will generalize all aspects for invasion ecology depicted in the MAFIA framework. It is most suitable to reflect similar co-transport scenarios in the real world, which hypothesized that alien species can act in synergy to amplify invasion impacts (e.g. pests or pathogens being transported by other non-native species [75, 76]). As phage interaction with non-host bacteria seems to be a widespread mechanism [19], future exploration of hyphae-associated phage-bacteria communities will not only resolve phage activities in regulating hyphosphere life, but also offer a powerful tool for testing hypotheses of invasion ecology at large scale (e.g. regarding spatial and trait relationships of alien biota) in spatially tailored microbial model systems.

## Supplementary information

Supplemental Material
