# Supplementary material for: Phage co-transport with hyphal-riding bacteria fuels bacterial invasion in a water-unsaturated microbial model system
Source: ISME J. 2021 Dec 13;16(5):1275–83. doi: 10.1038/s41396-021-01155-x (PMC9039081; doi:10.1038/s41396-021-01155-x)
Supplement: Supplementary file 1 — Supplemental Material [file 41396_2021_1155_MOESM1_ESM.pdf]

Supplementary information:

Phage co-transport with hyphal-riding bacteria fuels bacterial invasion in a water-unsaturated microbial model system

Xin You[1], René Kallies[1], Ingolf Kühn[2,3,4], Matthias Schmidt[5], Hauke Harms[1,4], Antonis Chatzinotas[1,4,6], Lukas Y. Wick[1,]*

[1] Helmholtz Centre for Environmental Research - UFZ, Department of Environmental Microbiology, Permoserstraße 15, 04318 Leipzig, Germany

[2] Helmholtz Centre for Environmental Research–UFZ, Department of Community Ecology, Theodor-Lieser-Str. 4, 06120 Halle, Germany

[3] Institute of Biology, Leipzig University, Talstr.33, Leipzig 04103, Germany

[4] German Centre for Integrative Biodiversity Research (iDiv) Halle-Jena-Leipzig, Deutscher Platz 5e, 04103 Leipzig, Germany

[5] Helmholtz Centre for Environmental Research–UFZ, Department of Isotope Biogeochemistry, Permoserstraße 15, 04318 Leipzig, Germany

[6] Institute of Biology, Leipzig University, Talstr.33, Leipzig 04103, Germany

Summary: The Supplementary information includes 9 pages and 5 figures

[*] Corresponding author. Phone: +49 341 235 1316, fax: +49 341 235 45 1316, e-mail: lukas.wick@ufz.de.

Extended Materials and Methods

Time-dependent transport rates (R_i), transport capacity (C_i) and transport efficiency (E_i) was used to evaluate phage-bacterial co-transport in microcosm setups shown in Fig. 2a. Eqs. S1 & S2 define the cumulative transport rates of bacteria (R_b, CFU cm^{-1} d^{-1}) and phages (R_p, PFU cm^{-1} d^{-1}) at given time (t, d). $N_{b,agarBC}$ (CFU) and $N_{p,agarBC}$ (PFU) reflect the number of infectious phages or bacteria on agar patches B & C, *d* (cm) the distance between inoculum and edge of the agar strip, and *t* (d) the time interval between inoculation and sampling. As T4 got rapidly deactivated on agar surfaces (99% loss of PFU in < 24 h, Fig. S2a) and subsequent low phage numbers were elusive to direct quantification, $N_{p,agarBC}$ (eq. S2) was approximated by the difference of phage numbers on agar patch A in the absence ($N_{p,agarA}$) and presence ($N'_{p,agarA}$) of carrier bacteria. This approach was possible as the presence of *P. ultimum* or *P. ultimum* and *P. putida* KT2440 co-cultures did not influence T4 infectivity (Fig. S2c).

$$R_b = \frac{N_{b,agarBC}}{d \times t} \qquad \text{eq. S1}$$

$$R_p = \frac{N_{p,agarBC}}{d \times t} \approx \frac{N_{p,agarA} - N'_{p,agarA}}{d \times t} \qquad \text{eq. S2}$$

The average number of phages co-transported by a single bacterial carrier, i.e. the apparent bacterial phage transport capacity (C_p, PFU bacteria^{-1}), is reflected by eq. S3.

$$C_p = \frac{N_{p,agarBC}}{N_{b,agarBC}} \approx \frac{N_{p,agarA} - N'_{p,agarA}}{N_{b,agarBC}} \qquad \text{eq. S3}$$

The fraction of phages dispatched by carrier bacteria, i.e. phage transport efficiency (E_p, %), was calculated by eq. S4.

$$E_p = \frac{N_{p,agarA} - N'_{p,agarA}}{N_{p,agarA}} \qquad \text{eq. S4}$$

Eq. S5 refers to the time-dependent absolute fitness (W_i) in order to reflect the effects of phage co-transport on the fitness of bacterial (W_b) or phage (W_p) populations in microcosm setups shown in Fig 3a. N_b (CFU) and N_p (PFU) represent the phage (i.e. T4) or bacteria (i.e. *P. putida* KT2440) counts on agar patches A, B or C in the absence of *E. coli*. N^*_b (CFU) and N^*_p (PFU) reflect the

phage (i.e. T4; N_{T4}) or bacteria (i.e. *P. putida* KT2440; N_{WT}) on agar patches A, B or C in presence *E. coli*. $W_i > 1$ and $W_i < 1$ indicate an increase and a decrease, resp. of the population size, while $W_i = 0$ refers to population extinction. At $t = 1$ d, no significant difference (P>0.05, Fig. 2d) between $N_{p,agarA}$ and $N'_{p,agarA}$ (i.e. an estimation for $N_{p,agarBC}$) was observed in the absence of *E.coli*. We thus assigned the PFU detection limit (= 200 PFU ml^{-1}) to $N_{p,agarBC}$ for a conservative estimation of W_p on day 1.

$$W_b = \frac{N^*_b}{N_b} \qquad W_p = \frac{N^*_p}{N_p} \qquad \text{eq. S5}$$

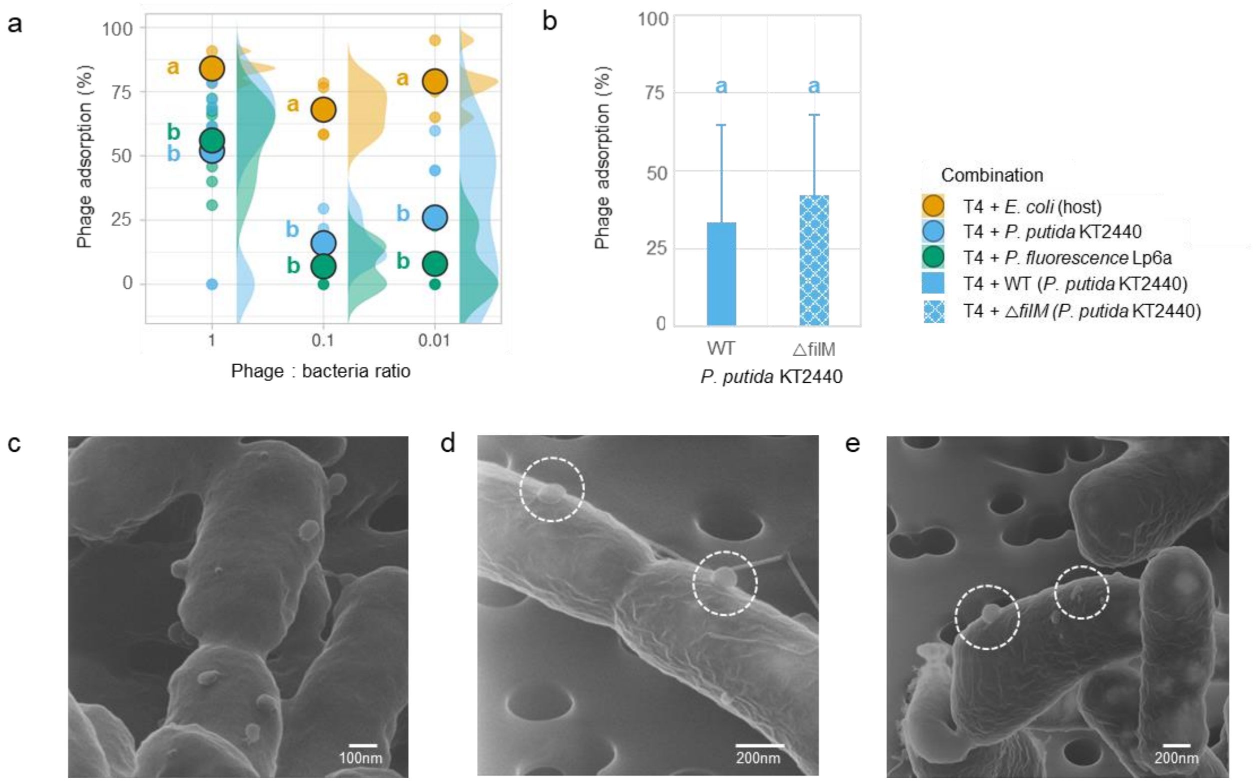

Figure S1. T4 adsorption to host *E. coli* bacteria, non-host bacteria *P. putida* KT2440 (WT and non-flagellated △*film*), and *P. fluorescence* Lp6a cells in liquid adsorption assays (cf. materials and methods); **a** Adsorption of T4 to *E. coli* host and non-host WT and *P. fluorescens* Lp6a at 3 tested phage to bacteria ratios (i.e. 0.01, 0.1 and 1). Data represent 6-8 replicates at each phage-to-bacteria ratio and different letters denote statistical significance at $P<0.05$ (Welch's *t* test); **b** Adsorption of T4 to flagellated WT and non-flagellated △*film* at a phage to bacteria ratio of 1. Different letters denote statistical significance at $P<0.05$ ($n = 3$; Welch's *t* test). **c** HIM visualization of T4 adsorption to *E.coli*. **d** & **e** HIM visualization of T4 adsorption to WT.

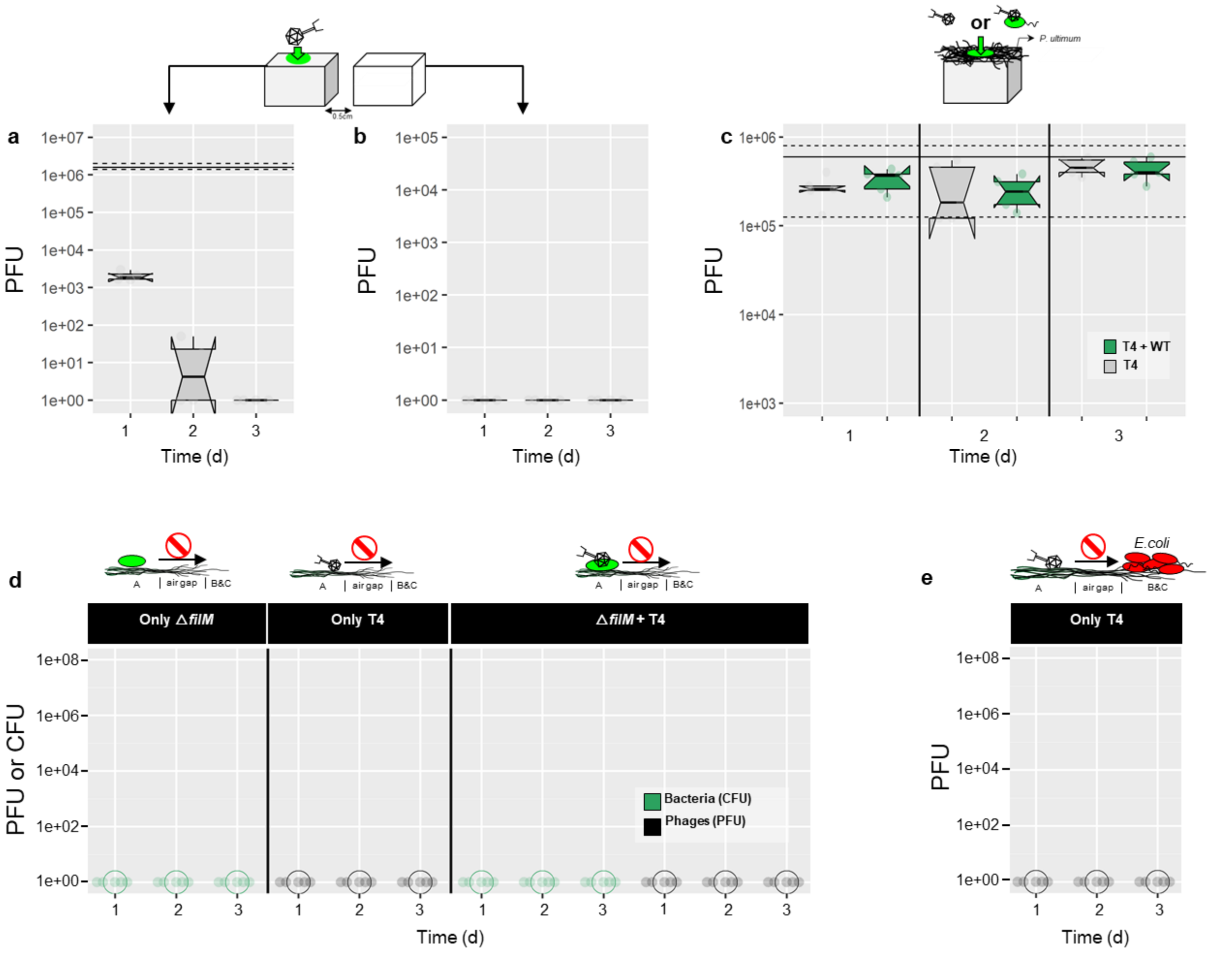

Figure S2. Time dependent abundance of infectious T4 on agar surfaces in presence and absence of additional biomass (*P. ultimum* or *P. ultimum* + *P. putida* KT2440) and diffusions of phages and non-motile *P. putida* KT2440 (△*filM*) along the hyphae with 3 days. **a&b** Decay of T4 on an agar surface over time (**a**; bold and dashed lines indicate the median ± 95% confidential interval of the inoculated amount; n = 5) and air-born transport of T4 to another agar surface at 0.5 cm distance (**b**). **c** Time-dependent decay of T4 on an agar surface overgrown either by *P. ultimum* or *P. ultimum* + *P. putida* KT2440 (bold and dash lines indicate the median ± 95% confidential interval of the inoculated amount; n = 5). **d** Diffusion of △*filM*, T4 and T4 adsorbed △*filM* (n=5) over the 0.5 cm distance in co-transport experiment in the absence of *E.coli* on agar B&C. **e** Diffusion of T4 (n=5) over the 0.5 cm distance in co-transport experiment in the presence of *E.coli* on agar B&C.

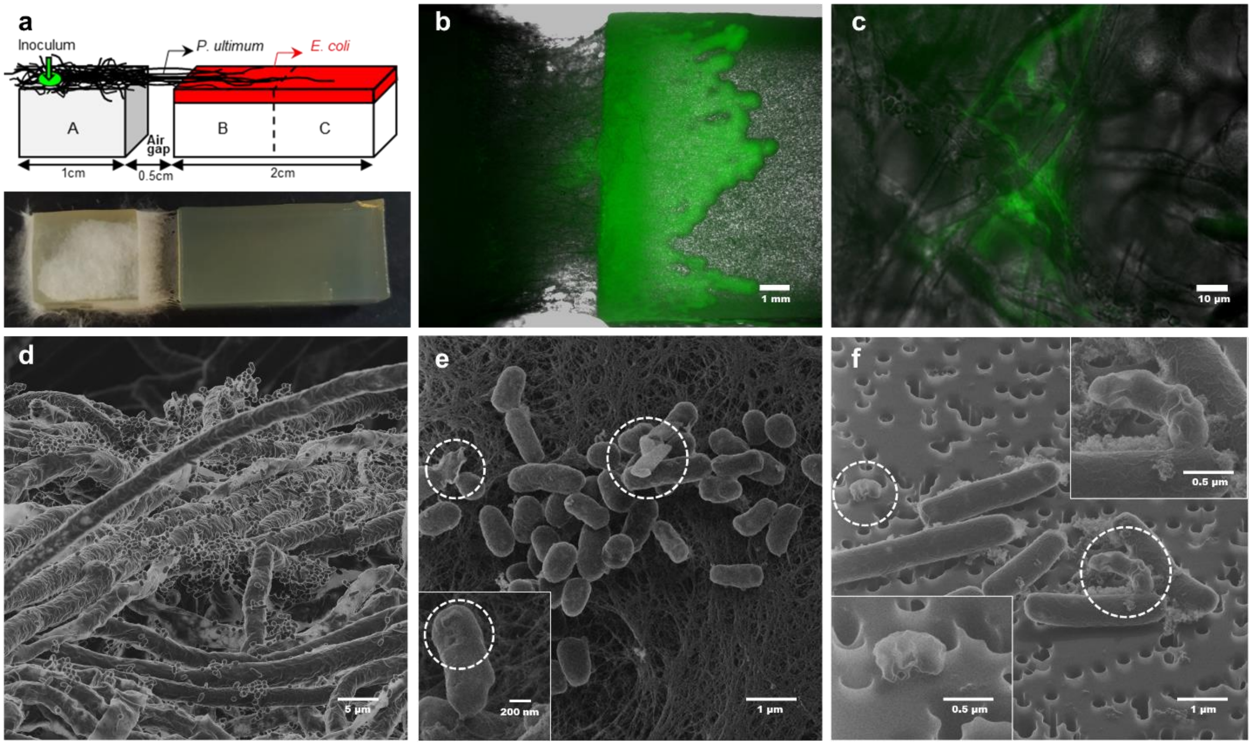

Figure S3. Microscopic visualization of T4 adsorbed *P. putida* KT2440 (WT) invading into an *E. coli* biofilm (**b&c**), *P. putida* KT2440 (WT) in the hyphosphere of *P. ultimum* (**d**), and T4 induced lysis of *E. coli* cells (**e&f**). **a** Scheme and top view photograph of the microcosm setup. **b** Representative fluorescence micrograph (60% opacity of gfp overlay) depicting the air-gap between agar patches A & B and invasion of T4 adsorbed and gfp-labelled WT (in green) to *E. coli* biofilm on agar patch B. **c** Representative fluorescence micrograph (60% opacity of gfp overlay) depicting establishment and growth of WT (in green) in the hyphosphere on agar patch C on day 3. **d - f** Helium ion micrographs visualizing E. coli and WT in the hyphosphere of *P. ultimum* (**d**) and lysed *E. coli* cells on agar patch B on day 2 (**e**) and of controls of T4 infected *E.coli* cells after 25 min of co-incubation with T4 in liquid medium (**f**).

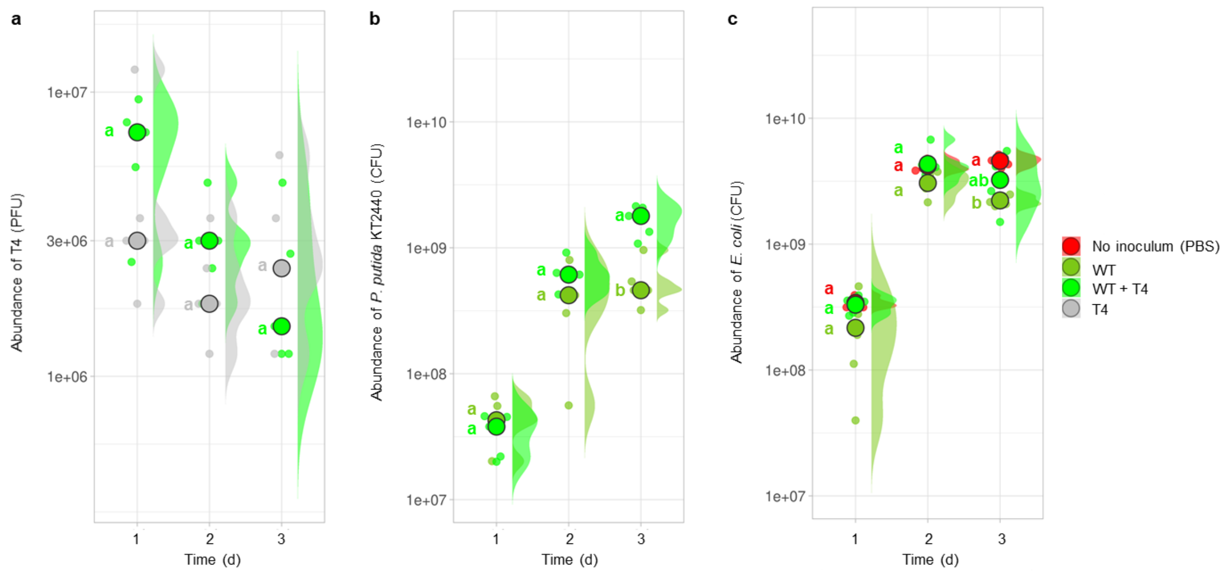

Figure S4. Abundances of T4, *P. putida* KT2440 (WT) and *E. coli* in the whole microcosm (agar patches A, B & C). **a** Abundances of T4 in the presence and absence of the WT carrier. **b** Abundances of WT in the presence and absence of T4. **c** Abundances of *E.coli* in the absence of WT and in presence of either WT or WT + T4. Data include 5 replicates and different letters denote statistical significance at $P < 0.05$ (Welch's t test).

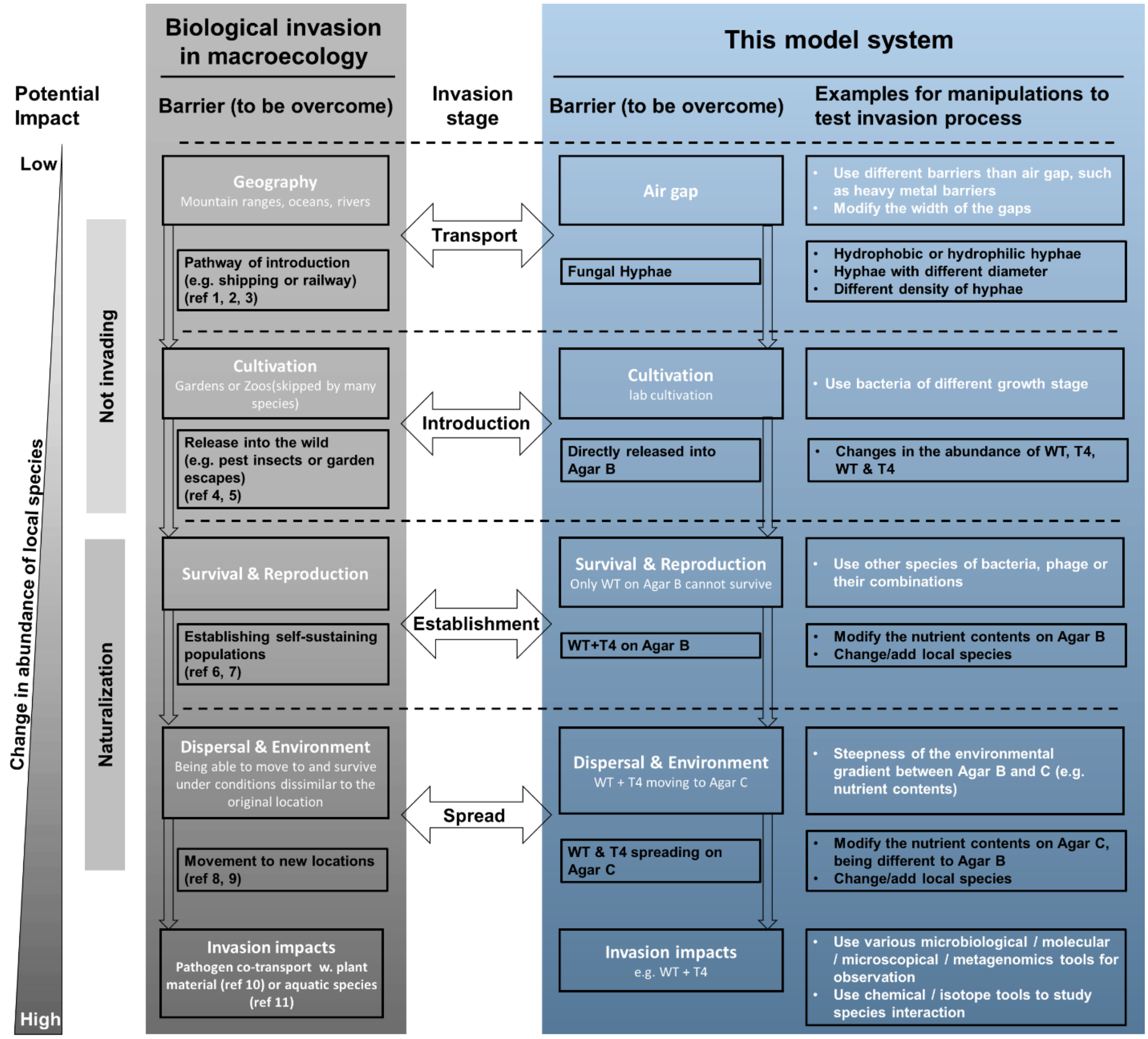

Figure S5. Comparison of important barriers (white fonts), processes by which they can be overcome (black fonts) and the respective stages (bi-directional arrows) of the invasion process of the frameworks with references (c.f. the "references" section at the end of the SI) to a few selected examples (left hand side in grey) and how this can be related to our proposed model system with examples for manipulations for each of the stages (right hand side in blue).

References

1. Seebens H, Gastner MT, Blasius B. The risk of marine bioinvasion caused by global shipping. Ecol Lett. 2013; 16: 782–790.

2. Seebens H, Essl F, Blasius B. The intermediate distance hypothesis of biological invasions. Ecol Lett. 2017; 20: 158–165.

3. Ascensão F, Capinha C. Aliens on the move: transportation networks and non-native species. In: Borda-de-Água L, Barrientos R, Beja P, Pereira HM (eds). Railway Ecology. 2017. Springer International Publishing, Cham, pp 65–80.

4. Pranty B. The budgerigar in florida: rise and fall of an exotic psittacid. North Am Birds. 2001; 55: 389–397.

5. The Bacchus marsh wattle, *Acacia rostriformis* (formerly *A. verniciflua*), see Blackburn TM, Pyšek P, Bacher S, Carlton JT, Duncan RP, Jarošík V, et al. A proposed unified framework for biological invasions. Trends Ecol Evol. 2011; 26: 333–339.

6. Kowarik I. Time lags in biological invasions with regard to the success and failure of alien species. Plant invasions Gen Asp Spec Probl. 1995; 15–38.

7. Ramírez-Albores JE, Richardson DM, Stefenon VM, Bizama GA, Pérez-Suárez M, Badano EI. A global assessment of the potential distribution of naturalized and planted populations of the ornamental alien tree Schinus molle. NeoBiota. 2021; 68: 105.

8. Keeling MJ, Franklin DN, Datta S, Brown MA, Budge GE. Predicting the spread of the Asian hornet (*Vespa velutina*) following its incursion into Great Britain. Sci Rep. 2017; 7: 1–7.

9. Hussner A, van de Weyer K, Gross EM, Hilt S. Comments on increasing number and abundance of non-indigenous aquatic macrophyte species in Germany. Weed Res. 2010; 50: 519–526.

10. Liebhold AM, Brockerhoff EG, Garrett LJ, Parke JL, Britton KO. Live plant imports: the major pathway for forest insect and pathogen invasions of the US. Front Ecol Environ. 2012; 10: 135–143.

11. Foster R, Peeler E, Bojko J, Clark PF, Morritt D, Roy HE, et al. Pathogens co-transported with invasive non-native aquatic species: implications for risk analysis and legislation. NeoBiota. 2021; 69: 79–102.
